# Lung Cancer Incidence Trends by Gender, Race and Histology in the United States, 1973–2010

**DOI:** 10.1371/journal.pone.0121323

**Published:** 2015-03-30

**Authors:** Rafael Meza, Clare Meernik, Jihyoun Jeon, Michele L. Cote

**Affiliations:** 1 Department of Epidemiology, University of Michigan, Ann Arbor, MI, United States of America; 2 Program in Biostatistics and Biomathematics, Fred Hutchinson Cancer Research Center, Seattle, WA, United States of America; 3 Department of Oncology, Wayne State University School of Medicine, Detroit, MI, United States of America; H. Lee Moffitt Cancer Center & Research Institute, UNITED STATES

## Abstract

**Background:**

Lung cancer (LC) incidence in the United States (US) continues to decrease but with significant differences by histology, gender and race. Whereas squamous, large and small cell carcinoma rates have been decreasing since the mid-80s, adenocarcinoma rates remain stable in males and continue to increase in females, with large racial disparities. We analyzed LC incidence trends by histology in the US with an emphasis on gender and racial differences.

**Methods:**

LC incidence rates from 1973–2010 were obtained from the SEER cancer registry. Age-adjusted incidence trends of five major histological types by gender and race were evaluated using joinpoint regression. Trends of LC histology and stage distributions from 2005–2010 were analyzed.

**Results:**

US LC incidence varies by histology. Squamous, large and small cell carcinoma rates continue to decrease for all gender/race combinations, whereas adenocarcinoma rates remain relatively constant in males and increasing in females. An apparent recent increase in the incidence of squamous cell carcinoma and adenocarcinoma since 2005 can be explained by a concomitant decrease in the number of cases classified as other non-small cell carcinoma. Black males continue to be disproportionally affected by squamous LCs, and blacks continue to be diagnosed with more advanced cancers than whites.

**Conclusions:**

LC incidence by histology continues to change over time. Additional variations are expected as screening becomes disseminated. It is important to continue to monitor LC rates to evaluate the impact of screening on current trends, assess the continuing benefits of tobacco control, and focus efforts on reducing racial disparities.

## Introduction

Despite the significant reductions of smoking during the last 50 years, lung cancer remains as the top cause of cancer-related death in the US, accounting for about 27.4% of all cancer deaths [1]. Lung cancer incidence has been decreasing for several years, particularly in males, however it is still the second most common cancer in both females and males, only behind breast and prostate cancer, respectively. Lung cancer incidence in the US is also characterized by significant racial disparities, with African American men having about 50% higher incidence than whites.

There are four major histological types of lung cancer; adenocarcinomas, squamous cell carcinomas, large cell carcinomas, and small cell carcinomas. In the past, bronchioalveolar carcinomas (BACs) were also considered as a separate histological type, while the current recommended system suggests categorizing these tumors with adenocarcinomas [2]. Although most lung cancers are due to smoking [3], the strength of the association and the corresponding attributable fraction vary greatly by histological type, with some types like small cell and squamous cell carcinomas thought to be almost exclusively due to smoking, and others like adenocarcinomas thought to be less dependent on smoking [4–7]. Moreover, important differences by histology exist in the smoking dose-relationships, and the latency times between exposure and outcome, as well as in the relative decrease in risk after smoking cessation [4–5,8–9]. Changes in cigarette composition have also affected the relative risks of lung cancer, likely also varying by histology [10–12]. All of this together with the decreases in smoking prevalence in the US since the 1950s [13] have led to significant changes in the lung cancer histology distribution, with adenocarcinomas overtaking squamous cell carcinomas as the most common type.

The epidemiology of lung cancer by histology is expected to continue to change as smoking rates decrease even further. In addition, the potential adoption of lung cancer screening as a tool for early detection may introduce additional alterations to the lung cancer histology distribution. The National Lung Cancer Screening Trial (NLST) recently demonstrated that low-dose Computed Tomography (LDCT) screening could effectively reduce lung cancer mortality among current and former heavy smokers [14]. The NLST findings together with extrapolations of the trial to the US population led the US Preventative Services Task Force (USPSTF) to recently revise their assessment and recommendation for lung cancer screening for at risk populations [15–16]. The USPSTF now recommends annual screening for lung cancer with low-dose computed tomography (LDCT) in adults aged 55 to 80 years who have a 30 pack-year smoking history and currently smoke or have quit within the past 15 years (grade B recommendation). Consequently, it is expected that lung cancer screening will become prevalent across the US in the next decade. Although screening is anticipated to have a positive impact in lung cancer mortality reduction [15–18] in common with other cancer screening modalities, it will also likely lead to overdiagnosis, i.e., to the detection of lung cancers that would not have otherwise been detected clinically [15–16,18]. Given that lung cancer overdiagnosis rates appear to vary greatly by histological type, with BACs being the type most susceptible to overdiagnosis [18], it is expected that screening will further affect the epidemiology of lung cancer by histological type.

We present joinpoint regression analyses of lung cancer incidence trends by histological type in the Surveillance, Epidemiology, and End Results (SEER) cancer registry, including BACs as a separate category due to the potential profound effects that screening may have on BAC overdiagnosis. Our analyses update and further quantify previous studies of lung cancer trends by histology in the US and elsewhere [8,19–26], and provide a detailed comparison of lung cancer incidence by race and gender in the US. These findings can be used as a baseline for future studies of the impact of smoking cessation and screening in the population.

## Materials and Methods

### Data

Malignant lung and bronchus cancer cases diagnosed between 1973 and 2010 were obtained from the nine original cancer registries in the SEER program of the National Cancer Institute (SEER-9): Atlanta, Connecticut, Detroit, Hawaii, Iowa, New Mexico, San Francisco-Oakland, Seattle-Puget Sound, and Utah [27]. SEER-9 was used in this analysis in order to examine registry data spanning the longest amount of time, as each registry in SEER-9 contributed cases diagnosed since 1973 (with the exception of Seattle-Puget Sound and Atlanta, which contributed cases since 1974 and 1975, respectively) [27]. The histology codes were grouped into five categories based largely on the International Agency for Research on Cancer (IARC) classifications [28]: (1) small cell carcinoma (International Classification of Diseases for Oncology Third Edition [ICD-O-3] codes 8041–8045, 8246]) (2) squamous cell carcinoma (8050–8078, 8083,8084); (3) large cell carcinoma (8012–8031, 8035, 8310); (4) adenocarcinoma (8140, 8211, 8230,8231, 8255–8260, 8323, 8480–8490, 8550,8551, 8570–8574, 8576); and (5) BAC (8250–8254). All lung and bronchus cancer histological types were also grouped together for general trend analysis (8000–9989). Race was categorized as white, black, or all—including white, black, American Indian, Alaskan Native, Asian/Pacific Islander.

### Analysis of trends

Age-adjusted incidence rates (AAIR) of lung and bronchus cancer (2000 U.S. standard population) per 100,000 person-years were calculated by gender, race, and histology using SEER*Stat software version 8.1.2 [29]. 95% confidence intervals (95% CI) were calculated using the Tiwari method [30].

Lung cancer incidence trends by gender, race and histology from 1973 to 2010 were evaluated using the Joinpoint Regression Program, version 4.0.4 [31]. Analyses were performed using the log-linear model (log-scale for rates) and allowed for a maximum of four joinpoints. Joinpoint regression analysis uses permutation tests to detect significant changes in the trend of incidence rates with 95% CI and selects the simplest number of joinpoints from the data. The annual percent change (APC) is calculated from the slope of the log-linear model at the segment between each joinpoint [32].

Relative proportions for the five histological types were calculated against all cases observed in these categories each year by gender and race. Relative incidence rates of cancer for blacks compared to whites and 95% confidence intervals [33] were calculated by gender and histology for 5-year intervals from 1973 to 2010 and for the entire period.

### Stage distribution

Gender-, race-, and histology-specific stage distributions for lung cancers diagnosed during 2005–2010 were estimated. Lung cancer cases were categorized as stage I, II, III, IV, or unknown based on the American Joint Committee on Cancer (AJCC) 6^th^ edition staging system.

## Results

### Descriptive analysis

**Fig 1:**
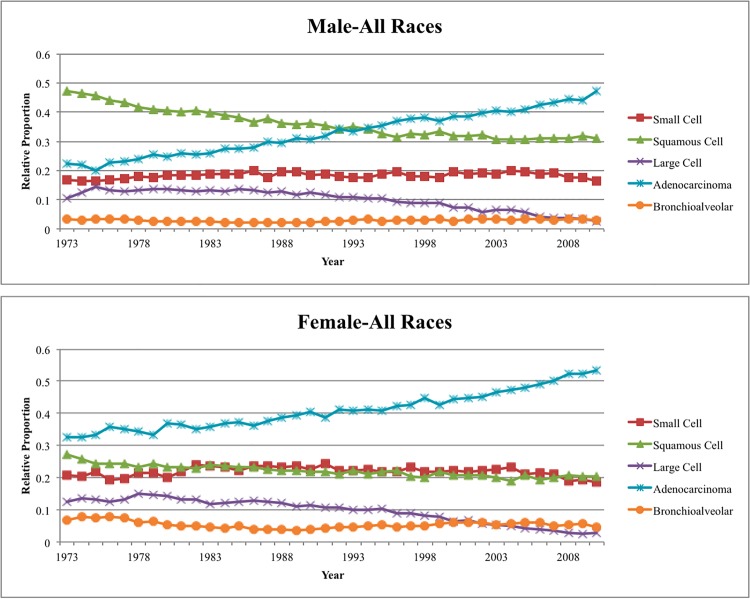
Relative proportions of lung and bronchus cancer cases in the United States SEER 9 registry by histology, 1973–2010.

Since 1973, squamous cell carcinomas have the highest incidence in males, followed by adenocarcinomas, small cell carcinomas, large cell carcinomas, and BACs. In contrast, adenocarcinomas have the highest incidence in females, followed by small cell carcinomas, squamous cell carcinomas, large cell carcinomas, and BACs. The age-adjusted rates stratified by calendar years are shown in S1 and S2 Tables. Fig. 1 shows the relative numbers of lung cancers by histology have changed dramatically since 1973. In males, squamous lung cancers were the most common and accounted for about half of lung cancers in males in the 1970s. Since that time, their relative numbers have decreased and now only account for about 30% of lung cancers. In contrast, adenocarcinomas currently account for over 40% of all lung cancer cases, whereas they were only about 20% of cases in 1973. The fraction of small cell carcinomas has remained roughly constant at 20%, whereas large cell carcinomas decreased from 10% to less than 3%. In females, adenocarcinomas have always been the most common type, but now account for more than 50% of cases in 2010 versus 30% of cases in 1973. The fraction of squamous cell carcinomas decreased from about 30% to 20% during this same period. Trends for small cell carcinoma and large cell carcinomas are similar to men. Similar figures for all gender/race combinations are shown in S1 Fig.

### Trend analysis

**Fig 2:**
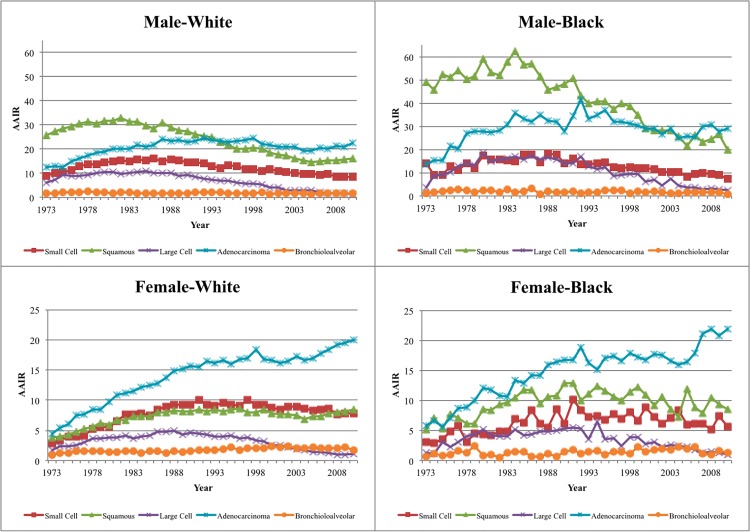
Age-adjusted incidence rates (AAIR) for lung and bronchus cancer cases in the US SEER 9 registry by histology, 1973–2010. Rate per 100,000 person-years (US Standard Population at year 2000).

Table 1, Fig. 2, and S2 Fig. show that the incidence of lung cancer in males of all races increased until 1980 and has been decreasing ever since, but patterns vary by histology. The incidence of small cell and large cell carcinomas increased until the mid-1980s and have been decreasing steadily since that time. Squamous cell carcinoma incidence increased until 1980, and then decreased significantly until 2005. The incidence of adenocarcinomas increased until the early 1990s, and then decreased until 2005. A non-significant increase in incidence starting in 2005, particularly in white males, is observed for squamous cell carcinoma and adenocarcinomas. BACs incidence remained roughly constant throughout the period of analysis, although a statistically significant decrease since the 1990s is observed. Blacks and whites follow similar trends in incidence, but blacks have relatively higher incidence of squamous cell carcinoma and adenocarcinoma (Incidence Rate Ratio [IRR] 1.67 and IRR 1.38, respectively), resulting in an overall higher incidence than white males (IRR 1.42).

**Table 1:** Trends in lung and bronchus cancer incidence rates in the United States SEER 9 registry in males by race and histology, 1973–2010.

	**Trend 1**		**Trend 2**		**Trend 3**		**Trend 4**	
	**Years**	**APC (95% CI)**	**Years**	**APC (95% CI)**	**Years**	**APC (95% CI)**	**Years**	**APC (95% CI)**
**All races**								
**Small Cell**	1973–1978	8.1* (6, 10.2)	1978–1986	2.1* (0.9, 3.3)	1986–2010	−2.7* (−2.9, −2.5)		
**Squamous Cell**	1973–1981	2.6* (1.8, 3.5)	1981–1990	−2.0* (−2.9, −1.2)	1990–2005	−4.0* (−4.3, −3.6)	2005–2010	0.9 (−0.8, 2.7)
**Large Cell**	1973–1975	25.2* (3.4, 51.5)	1975–1987	1.3* (0, 2.6)	1987–1998	−5.8* (−7.2, −4.4)	1998–2010	−11.0* (−12, -10)
**AC**	1973–1979	8.4* (6.4, 10.3)	1979–1992	2.2* (1.5, 2.8)	1992–2005	−1.7* (−2.3, −1.1)	2005–2010	2.6* (0.2, 5.1)
**BAC**	1973–1976	9.3 (−3.2, 23.4)	1976–1988	−3.0* (−4.5, −1.4)	1988–1993	4.8 (−2.9, 13.2)	1993–2010	−1.7* (−2.5, −0.9)
**All Histologies**	1973–1980	2.2* (1.6, 2.8)	1980–1991	−0.3 (−0.6, 0.1)	1991–2010	−1.8* (−2, −1.7)		
**White**								
**Small Cell**	1973–1978	8.3* (6.1, 10.6)	1978–1986	2.1* (0.8, 3.4)	1986–2010	−2.6* (−2.8, −2.4)		
**Squamous Cell**	1973–1980	3.0* (1.9, 4.1)	1980–1988	−1.3* (−2.4, −0.2)	1988–2005	−3.9* (−4.2, −3.5)	2005–2010	1.5 (−0.4, 3.4)
**Large Cell**	1973–1975	23.5* (4.1, 46.6)	1975–1986	1.6* (0.2, 2.9)	1986–1998	−5.3* (−6.4, −4.2)	1998–2010	−11.1* (−12, −10.2)
**AC**	1973–1980	7.2* (5.6, 8.9)	1980–1992	2.0* (1.2, 2.8)	1992–2005	−1.6* (−2.3, −1)	2005–2010	2.1 (−0.5, 4.7)
**BAC**	1973–1976	9 (−3.6, 23.3)	1976–1989	−2.5* (−3.9, −1.1)	1989–1992	7.9 (−15.6, 38.1)	1992–2010	−1.5* (−2.3, -0.7)
**All Histologies**	1973–1978	2.7* (1.8, 3.6)	1978–1989	0.2 (−0.1, 0.5)	1989–2010	−1.7* (−1.8, −1.6)		
**Black**								
**Small Cell**	1973–1986	4.1* (2.2, 6)	1986–2010	−3.1* (−3.8, −2.4)				
**Squamous Cell**	1973–1984	2.0* (0.5, 3.5)	1984–2010	−3.8* (−4.2, −3.4)				
**Large Cell**	1973–1977	33.5* (16.8, 52.6)	1977–1992	0.2 (−1.7, 2.2)	1992–2010	−9.7* (−10.9, −8.5)		
**AC**	1973–1979	13.5* (8.9, 18.2)	1979–1994	1.6* (0.4, 2.7)	1994–2004	−3.3* (−5.4, −1.1)	2004–2010	2.4 (−1.7, 6.7)
**BAC**	1973–2010	−1.0* (−1.9, 0.1)						
**All Histologies**	1973–1986	2.4* (1.6, 3.2)	1986–2010	−2.2* (−2.5, -1.9)				

*Annual percent change (APC) is significantly different from zero at α = 0.05.

APC based on incidence rates per 100,000 person-years.

AC, adenocarcinoma, BAC, bronchioloalveolar.

In contrast, female lung cancer incidence increased until 2007, although progressively at a lower annual percentage change (APC) rate. Since then, lung cancer incidence has been decreasing at an APC of −2.6 (95% Confidence Interval (CI): −4.8, −0.4) (Table 2). The trends vary greatly by histology. Small cell carcinoma incidence increased until the early 1990s and has been decreasing ever since. Large cell carcinoma incidence followed a similar pattern, although with a very significant decrease since 1997 (APC = −9.9, 95% CI: −10.7, −9.2). Squamous cell carcinomas increased until 1988, remained roughly constant until 1999, decreased from 1999 until 2004, and have been increasing since that time. Adenocarcinomas increased until 1991, remained roughly constant until 2004, when they again started increasing. BACs incidence remained roughly constant throughout the period, but with some variations by race (S3 Fig.). Patterns by race are roughly consistent, although overall incidence in black females is not yet decreasing. Black females have an overall higher incidence than white females (IRR 1.09), which is largely driven by their higher rates of squamous cell carcinomas (IRR 1.34) and adenocarcinomas (IRR 1.09).

**Table 2:** Trends in lung and bronchus cancer incidence rates in the United States SEER 9 registry in females by race and histology, 1973–2010.

	**Trend 1**		**Trend 2**		**Trend 3**		**Trend 4**		**Trend 5**	
	**Years**	**APC (95% CI)**	**Years**	**APC (95% CI)**	**Years**	**APC (95% CI)**	**Years**	**APC (95% CI)**	**Years**	**APC (95% CI)**
**All races**										
**Small Cell**	1973–1982	9.9* (8.7, 11.1)	1982–1991	3.3* (1.9, 4.6)	1991–2010	−1.3* (−1.7, −1)				
**Squamous Cell**	1973–1981	6.8* (5.9, 7.7)	1981–1988	3.2* (1.8, 4.5)	1988–1999	−0.3 (−0.9, 0.3)	1999–2004	−3.3* (−5.6, −0.9)	2004–2010	2.4* (1.1, 3.8)
**Large Cell**	1973–1978	13.7* (9.7, 17.8)	1978–1988	2.7* (1.2, 4.2)	1988–1997	−2.8* (−4.5, −1.1)	1997–2010	−9.9* (−10.7, −9.2)		
**AC**	1973–1976	15.1* (10.8, 19.5)	1976–1981	7.7* (5.1, 10.3)	1981–1991	4.4* (3.7, 5.2)	1991–2004	0.2 (−0.3, 0.6)	2004–2010	3.3* (2, 4.6)
**BAC**	1973–1976	16.1* (6.3, 26.7)	1976–1989	−1.5* (−2.5, −0.4)	1989–1992	10.8 (−7, 32)	1992–2001	1.9* (0, 3.9)	2001–2010	−1.4 (−3, 0.2)
**All Histologies**	1973–1976	9.0* (6.6, 11.5)	1976–1983	5.1* (4.3, 5.9)	1983–1991	3.4* (2.8, 4)	1991–2007	0.5* (0.3, 0.7)	2007–2010	−2.6* (−4.8, −0.4)
**White**										
**Small Cell**	1973–1982	10.4* (9.1, 11.7)	1982–1991	3.3* (1.8, 4.8)	1991–2010	−1.1* (−1.5, −0.7)				
**Squamous Cell**	1973–1979	8.5* (7, 10)	1979–1988	3.6* (2.7, 4.5)	1988–1999	−0.1 (−0.7, 0.5)	1999–2004	−3.0* (−5.5, −0.5)	2004–2010	3.0* (1.6, 4.4)
**Large Cell**	1973–1978	14.1* (9, 19.4)	1978–1988	3.0* (1.1, 4.9)	1988–1997	−2.6* (−4.8, −0.4)	1997–2010	−10.1* (−11, −9.1)		
**AC**	1973–1976	19.2* (13.8, 24.9)	1976–1988	5.9* (5.3, 6.6)	1988–1998	1.6*(0.7, 2.4)	1998–2001	−3 (−11.7, 6.4)	2001–2010	2.5* (1.6, 3.4)
**BAC**	1973–1976	17.5* (6.6, 29.4)	1976–1987	−0.8 (−2.3, 0.8)	1987–2000	3.8* (2.6, 5)	2000–2010	−1.5* (−3, 0)		
**All Histologies**	1973–1976	10.3* (7.4, 13.3)	1976–1988	4.7* (4.3, 5.1)	1988–1997	1.4* (0.8, 2)	1997–2007	0.3 (−0.2, 0.7)	2007–2010	−2.3 (−4.9, 0.4)
**Black**										
**Small Cell**	1973–1991	5.3* (3.7, 6.9)	1991–2010	−1.6* (−2.9, −0.2)						
**Squamous Cell**	1973–1990	4.6* (3.2, 6)	1990–2010	−1.8* (−2.8, −0.8)						
**Large Cell**	1973–1978	29.1* (14.6, 45.5)	1978–1994	0.9 (−1.4, 3.2)	1994–2010	−8.4* (−10.2, −6.5)				
**AC**	1973–1980	9.9* (7, 12.8)	1980–1990	4.6* (2.7, 6.5)	1990–2005	0.2 (−0.8, 1.1)	2005–2010	5.9* (1.3, 10.7)		
**BAC**	1973–2010	1.6* (0.5, 2.7)								
**All Histologies**	1973–1990	4.6* (4, 5.3)	1990–2010	0.3 (-0.2, 0.7)						

*Annual percent change (APC) is significantly different from zero at α = 0.05.

APC is based on incidence rates per 100,000 person-years.

AC, adenocarcinoma; BAC, bronchioloalveolar.

Relative incidence rates in blacks versus whites by calendar year are shown in S4 Fig. Overall the relative incidence in blacks versus whites hasn't changed much since the 1970s (Male IRR: 1.4 vs 1.37 and Female IRR: 1.12 vs 1.04 in 1976–1980 and 2006–2010, respectively). However the relative incidence of squamous cell carcinoma decreased (Male IRR: 1.73 vs 1.53 and Female IRR: 1.31 vs 1.13 in 1976–1980 and 2006–2010, respectively), whereas the relative incidence of large cell carcinoma increased (Male IRR: 1.45 vs 1.76 and Female IRR: 1.12 vs 1.17 in 1976–1980 and 2006–2010, respectively).

**Fig 3:**
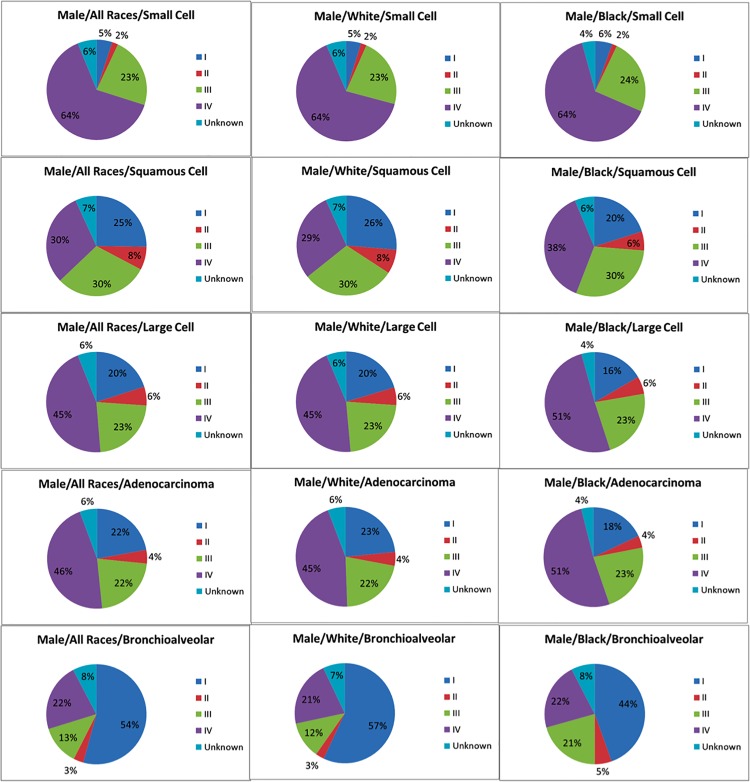
Stage distribution of male lung and bronchus cancer cases in the United States SEER 9 registry by race and histology, 2005–2010.

Fig. 3 shows the stage distributions by race and histology for male lung cancers diagnosed between 2005–2010. The figure shows that most small cell carcinomas are detected in stage IV (64%), whereas most BACs are detected in stage I (54%). For other histological types, about 60% or more are detected in stages III and IV. The figure also shows that for most histological types, blacks tend to be diagnosed with more advanced cancers (III/IV) than whites (squamous cell carcinoma 68% vs 59%, large cell carcinoma 74% vs 68%, adenocarcinoma 74% vs 67%, BAC 43% vs 33%). A similar pattern is shown in the stage distributions for female cancers (S5 Fig.).

## Discussion

### Main findings

We provide a current trend analysis and detailed description of the epidemiology of lung cancer in the US by histological type, gender and race, utilizing joinpoint regression to quantify the annual percent change of trends. Overall, lung cancer incidence continues to decrease following the significant decreases in smoking in the US since the 1960s [13]. However, incidence trends vary greatly by histology, with adenocarcinomas still continuing its upward (or non-decreasing trend), while the incidence of other histological types—small cell, squamous cell and large cell carcinomas—continues to decrease, as has been previously reported [2,10,19,24]. This has resulted in the continuous growth in the proportion of adenocarcinomas, which are now the most commonly diagnosed histological type in both women and men. A recent (since 2004/2005) increase in the incidence rates of squamous cell carcinomas and adenocarcinomas, particularly in whites, can be attributed to a concomitant decrease in cancers classified as “other non-small cell lung cancer,” likely due to the need for better histological classification in response to the development of new therapies that are histology specific [2,10,26,34–35].

Racial disparities in lung cancer incidence continue to exist, with blacks, particularly black men, having significantly higher lung cancer rates than whites. Nonetheless, the black versus white relative incidence rate of squamous cell carcinomas appears to be now decreasing. With regards to stage, blacks continue to be diagnosed with more advanced cancers than whites independent of histology, explaining in part the well known racial disparities in lung cancer mortality in the US [36–37]. As screening becomes more readily available, it will be important to monitor lung cancer incidence and stage at diagnosis by race and gender, so that resources can be put in place to identify the groups that may need targeted screening efforts.

### Strengths and limitations

In common with any analysis of cancer registry data, our study has several limitations. First, being an ecological study, it is not possible to investigate causal relationships between the observed trends and relevant lung cancer risk factors, like smoking, asbestos exposures, or socioeconomic status. Moreover, SEER lacks information about the smoking history of lung cancer cases, which precludes analyses of lung cancer trends and histology distributions by smoking status. Second, histological classifications are continuously evolving, making it difficult to untangle true changes in incidence versus changes due to improvements or variations in disease classification. Nonetheless, the SEER cancer registry provides histology and stage classifications adjusted for historical changes in clinical definitions, minimizing the impact of such artificial secular effects. Lastly, we were unable to examine trends by Hispanic ethnicity using the SEER data, which started collecting this information in 1992.

Our study also has several strengths. We used almost 40 years of lung cancer incidence data from arguably the best cancer registry in the world covering about 10% of the US population. Thus our analyses had sufficient statistical power to distinguish differences in lung cancer incidence by race and gender for all histological categories evaluated. Moreover, the joinpoint regression methodology allows for objective analyses of incidence trends, therefore allowing for direct comparison of the estimated trends between distinct epidemiological groups. While most previous analyses extended until the late 1990s, our study includes all years available in the SEER registry, through 2010 [2,19,24]. Our study also extends previous analyses by providing relative incidence estimates of lung cancer trends and stage distributions by histology between whites and blacks in the US, demonstrating that racial disparities observed since the 1970s continue to persist in the 21st century.

### Implications and future research

Our analyses provide a detailed description of the current status of lung cancer epidemiology in the US. Current lung cancer incidence rates are expected to continue to decrease in response to further reductions in smoking prevalence, particularly among females. If trends continue, we would expect to see additional decreases in the incidence of histological types strongly dependent on smoking, notably small cell, squamous cell and large cell carcinomas, while other types, notably adenocarcinomas, may remain constant or continue to increase, at least proportionally. Further studies characterizing differences in smoking dose response by histology, and on the associations of traditional lung cancer risk factors and other covariates with lung cancer by histological type are necessary to better understand current trends, particularly in non-smokers [38–39].

The potential widespread adoption of lung cancer screening in response to the recent USPSTF screening recommendations is likely to induce further fluctuations in the relative incidence and frequency of different lung cancer histological types. It appears that screening is particularly effective in the early detection and successful treatment of adenocarcinomas and BACs, and thus we could expect temporary increases in incidence, particularly of such histological types, once screening is disseminated across the US (a potential positive note, given the plausible synergies between the increase in prevalence of adenocarcinomas and the efficacy of screening in reducing adenocarcinoma mortality) [14,40]. Related to this is the likelihood that screening will lead to the diagnosis of cancers that would have not otherwise become clinically detected (overdiagnosis). Lung cancer overdiagnosis rates due to CT screening appear to vary greatly by histology [18], with particularly high rates for BACs. Thus, it is plausible that screening will induce further artificial changes in the histology distributions. For all of these reasons, it will be important to continue to monitor in detail lung cancer incidence and mortality trends and the corresponding stage distributions by histological type as screening is disseminated in the US. The ability to monitor such trends by smoking history would be ideal. Moreover, future analyses of lung cancer trends in the US will need to take into account the potential changes in detection rates due to screening. Tracking the progressive uptake rates of screening and the fraction of lung cancers detected by screening will help researchers to gauge the impact of screening on incidence rates.

Finally, our analyses demonstrate that racial disparities in lung cancer rates in the US continue to exist, and that despite the significant decreases in incidence across all racial groups, the higher relative incidence rates in blacks remain, and that blacks continue to be disproportionately diagnosed with advanced lung cancers. This highlights the critical need for further studies that allow for proper characterization of differences in lung cancer risk and risk factor exposure in the US by race and ethnicity [5,41–43]. As lung cancer screening becomes disseminated, it is important to be vigilant so that potential racial and ethnic differences in screening access and uptake do not exacerbate existing disparities in lung cancer incidence and mortality outcomes albeit reducing the overall mortality risk in the whole population. An effort to effectively disseminate screening among those eligible, while limiting uptake in low-risk individuals, across all racial and ethnic groups is paramount.

## Supporting Information

S1 Fig Relative proportions of lung and bronchus cancer cases in the United States SEER 9 registry by histology, 1973–2010. (TIFF)

S2 Fig Age-adjusted incidence^a^ (AAIR) for lung and bronchus cancer cases in the United States SEER 9 registry by histology, 1973–2010. (TIFF)

S3 Fig Annual Percent Change (APC) Joinpoint plots for all lung and bronchus cancer cases in the United States SEER 9 registry by gender, race, and histology, 1973–2010. APC based on incidence rates per 100,000 person-years (^ indicates APC is significantly different from zero at α = 0.05).
(DOC)

S4 Fig The relative risks of cancer for blacks compared to whites in the United States SEER 9 registry by gender and histology, 1973–2010. (TIFF)

S5 Fig Stage distribution of female lung and bronchus cancer cases in the United States SEER 9 registry by race and histology, 2005–2010. (TIFF)

S1 Table Age-adjusted incidence rates^a^ (AAIR) of all lung and bronchus cancer cases reported to the United States SEER 9 registry by race and histology, 1973–2010. ^a^Rate per 100,000 person-years (US Standard Population at year 2000) with 95% confidence intervals obtained using the Tiwari method. Rows are grouped by roughly 10-year time periods.
(DOC)

S2 Table Age-adjusted incidence rates^a^ (AAIR) of all lung and bronchus cancer cases reported to the United States SEER 9 registry by race and histology, 1973–2010. ^a^Rate per 100,000 person-years (US Standard Population at year 2000) with 95% confidence intervals obtained using the Tiwari method. Rows are grouped by roughly 10-year time periods.
(DOC)
