# Supplementary figures and images for: Lung Cancer Incidence Trends by Gender, Race and Histology in the United States, 1973–2010
Source: PLoS One. 2015 Mar 30;10(3):e0121323. doi: 10.1371/journal.pone.0121323 (PMC4379166; doi:10.1371/journal.pone.0121323)

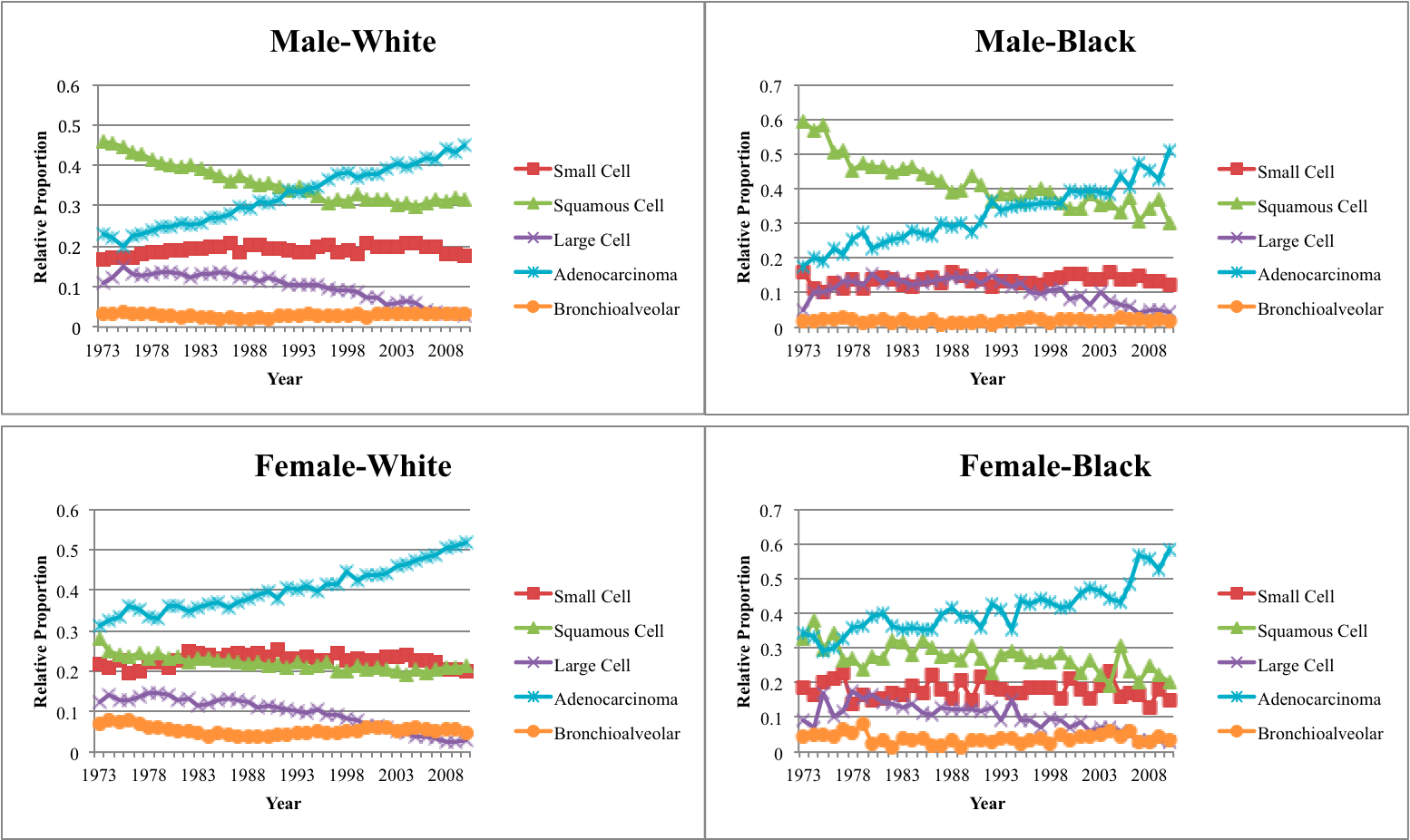

Supplement: S1 Fig — (TIFF) [file pone.0121323.s001.tiff]

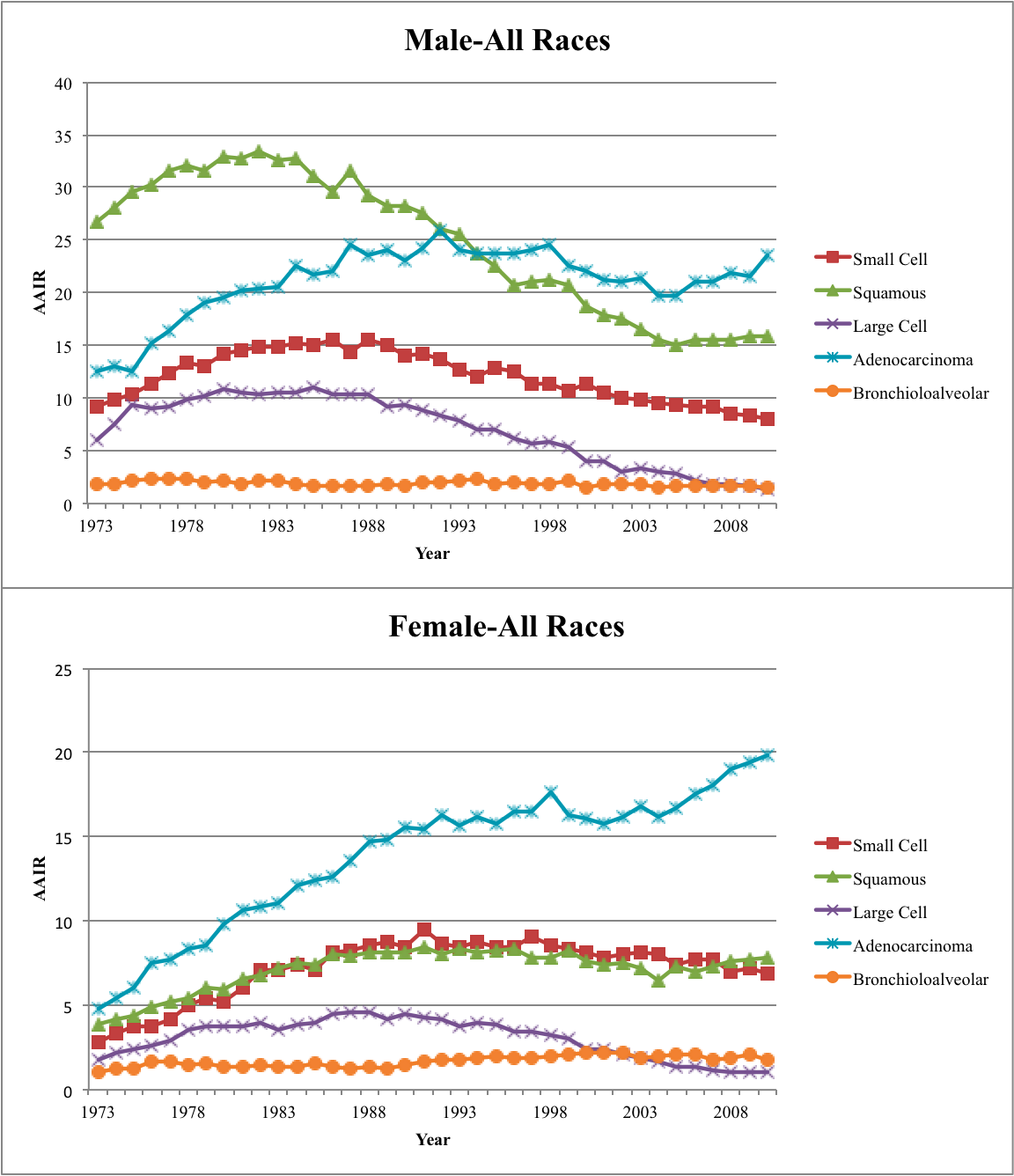

Supplement: S2 Fig — (TIFF) [file pone.0121323.s002.tiff]

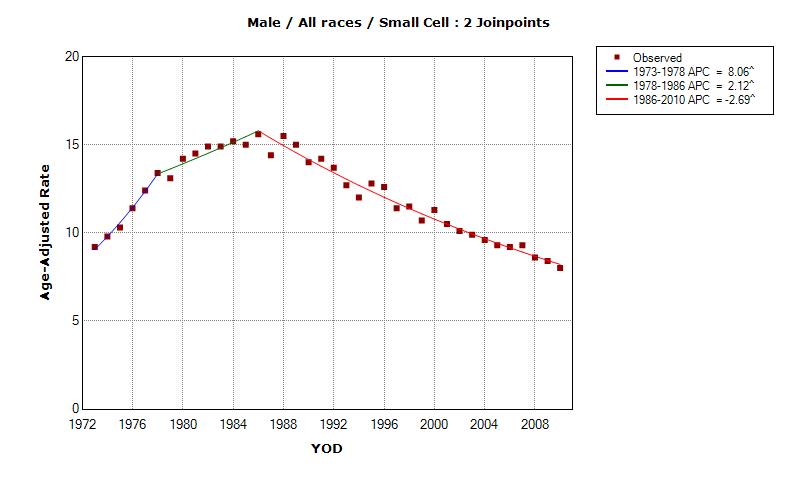

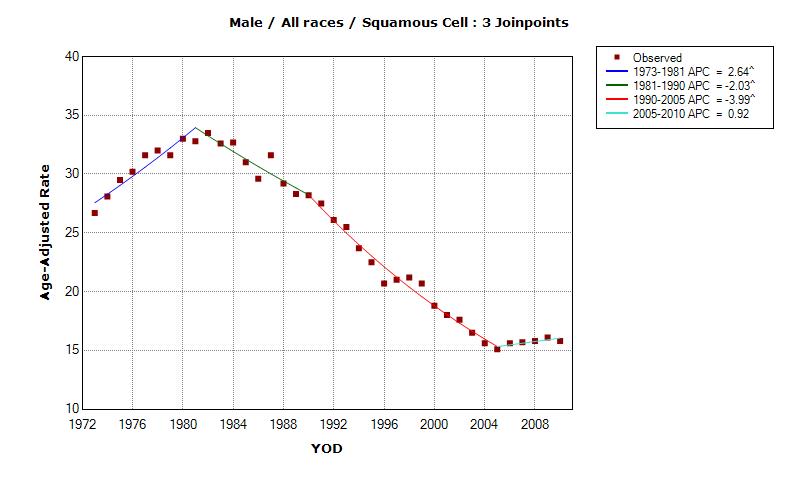

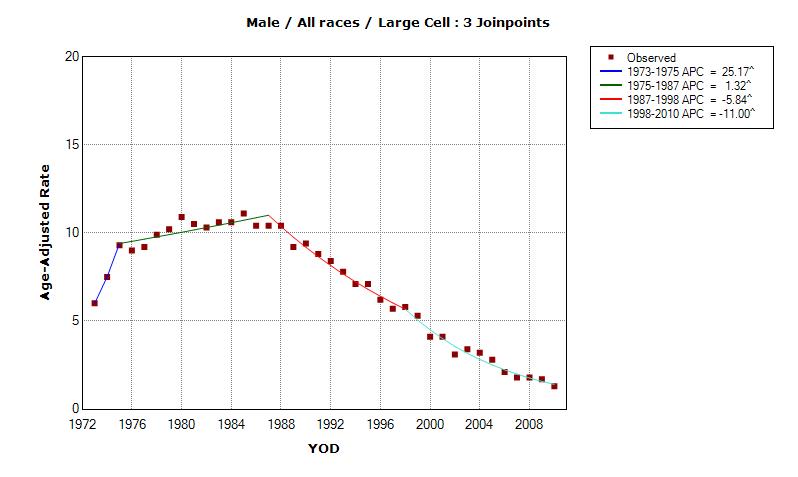

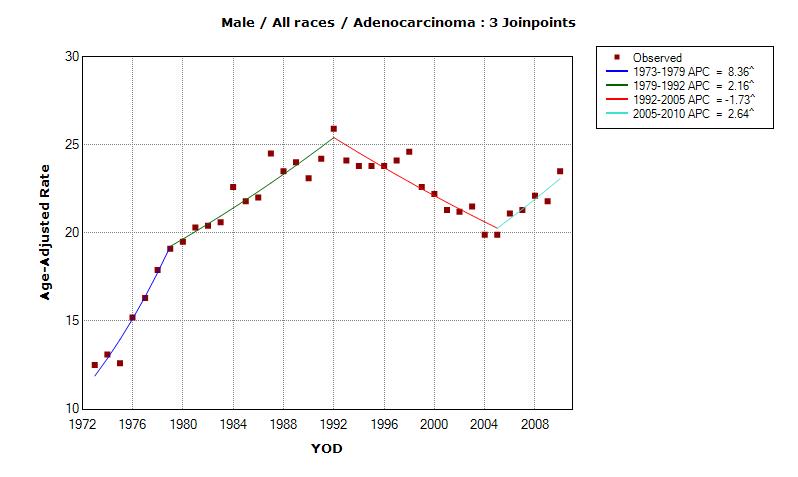

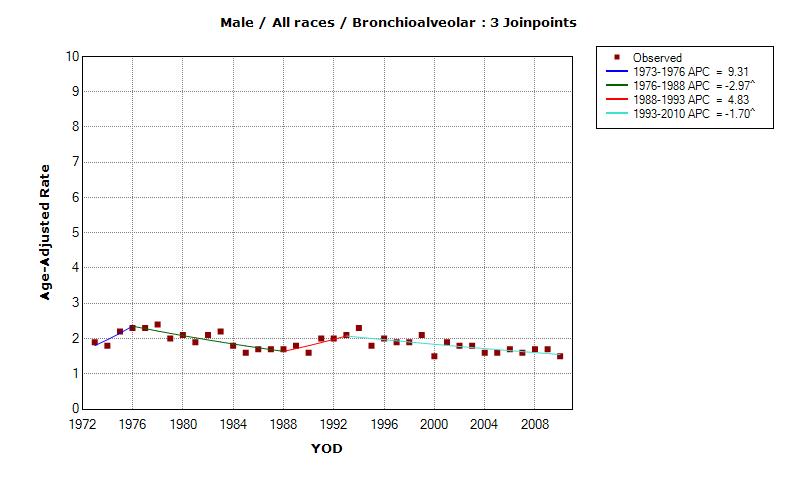


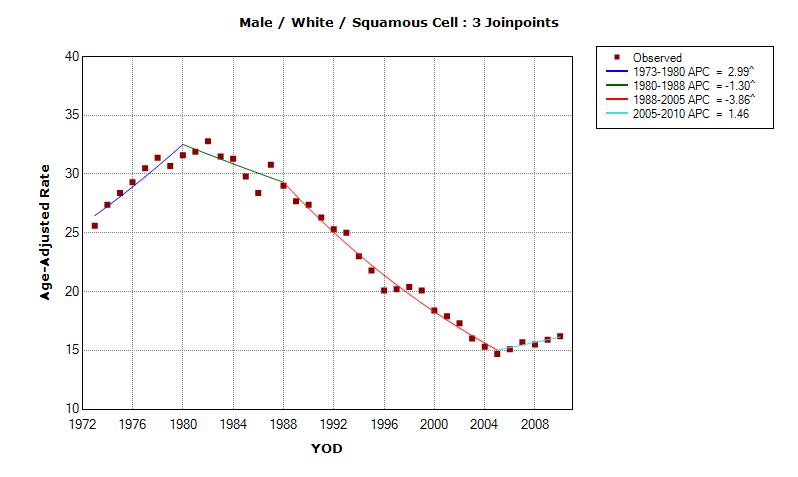


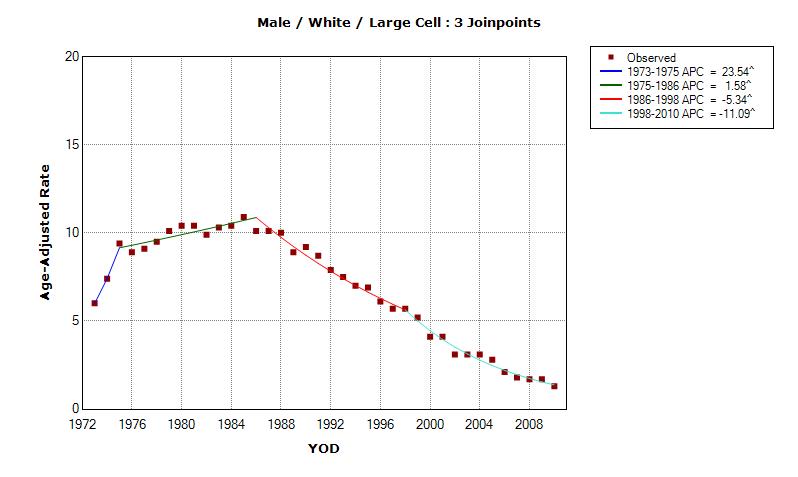


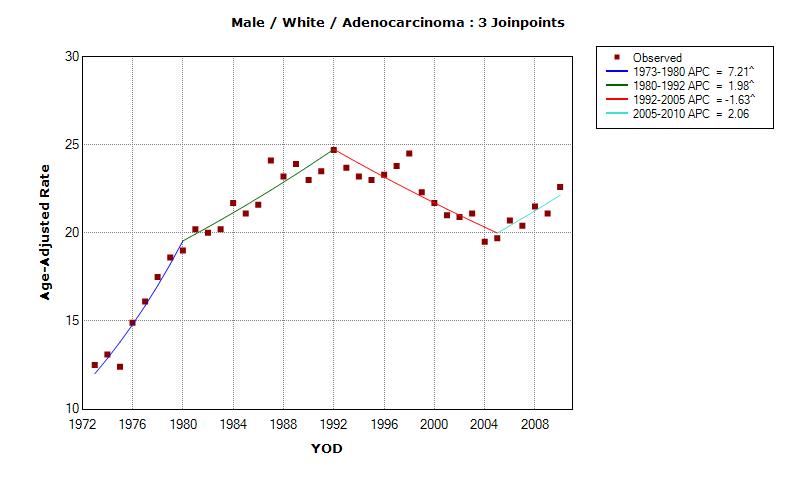


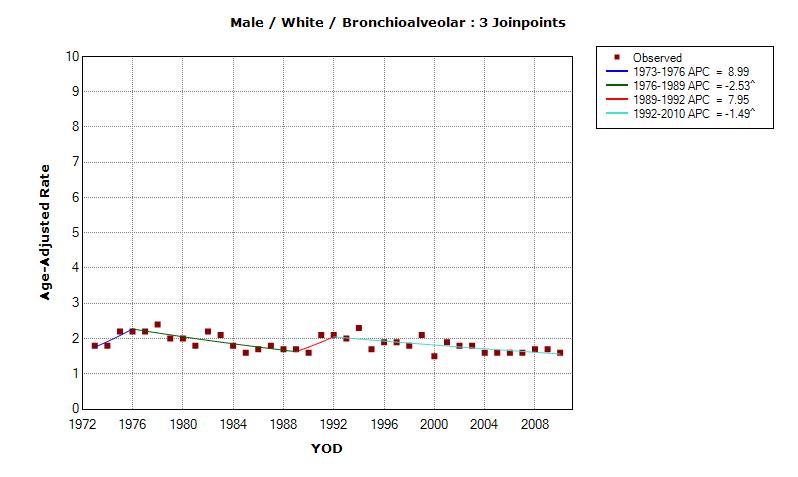


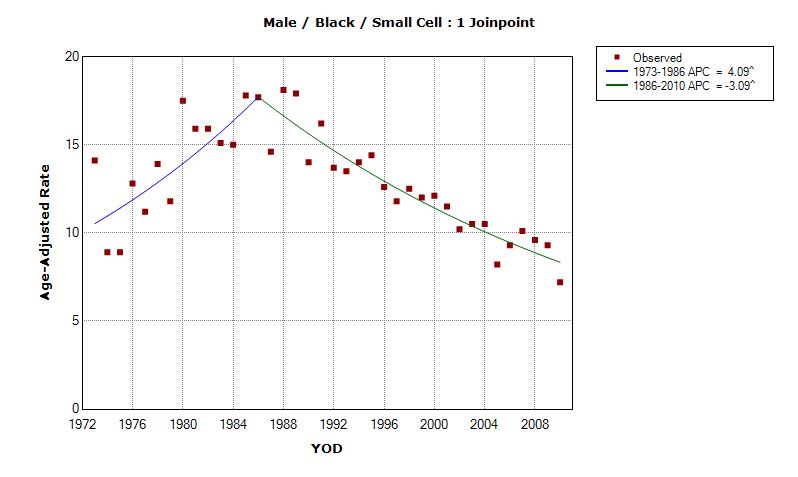

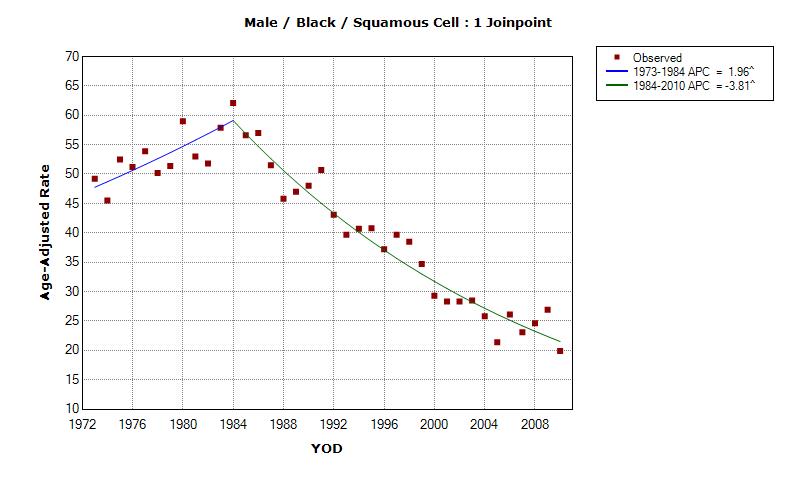

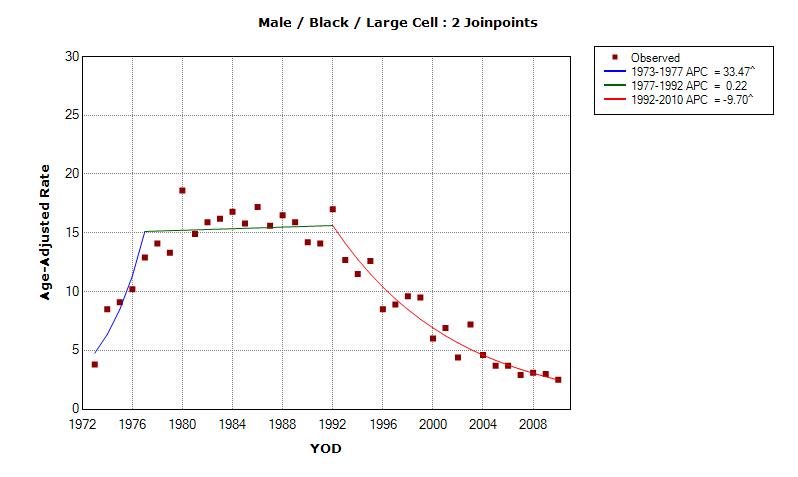

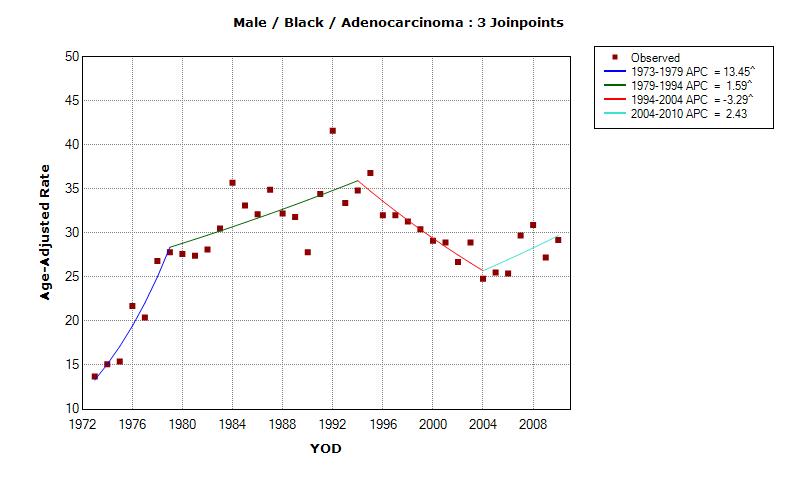

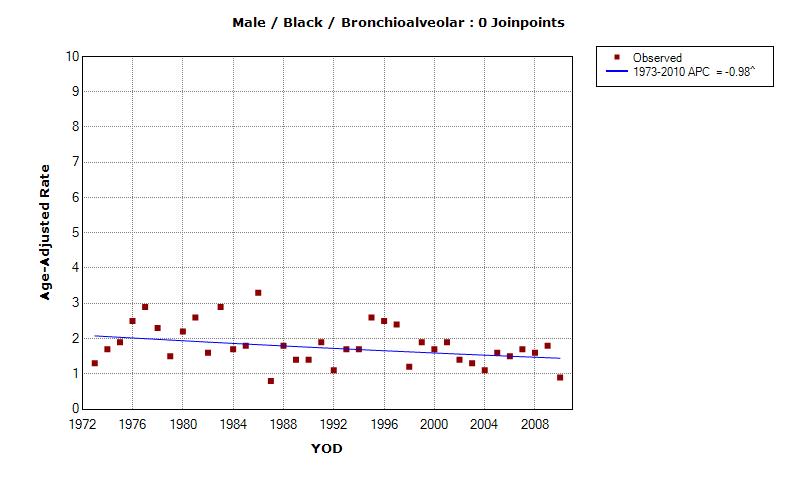

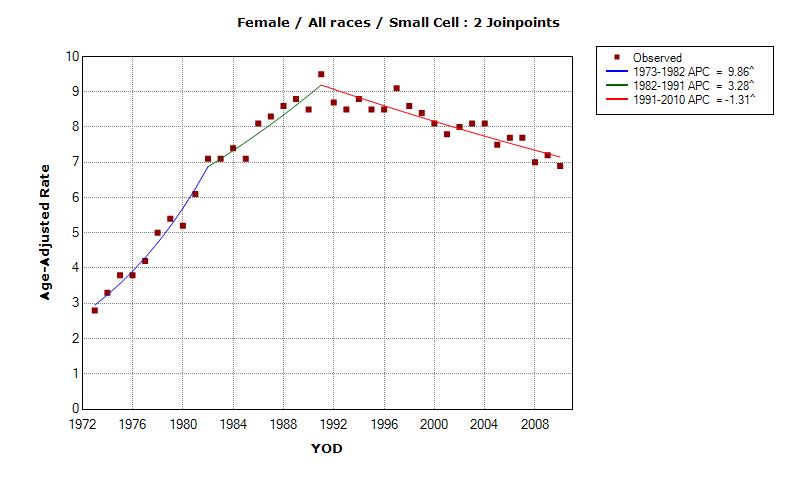


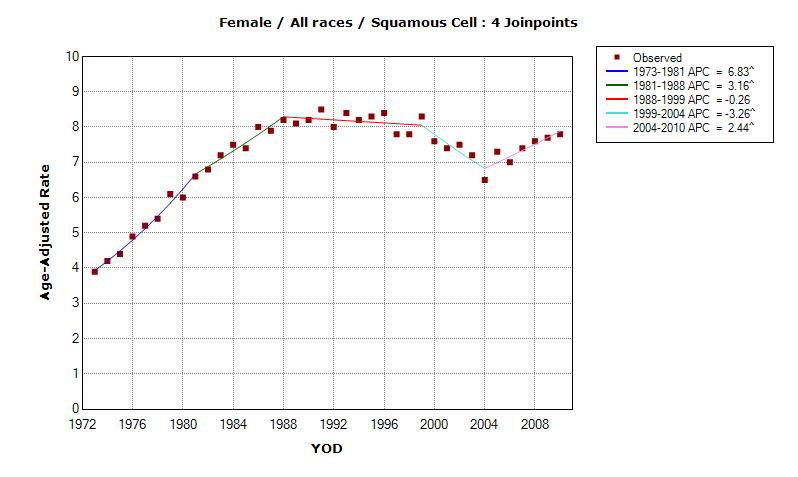

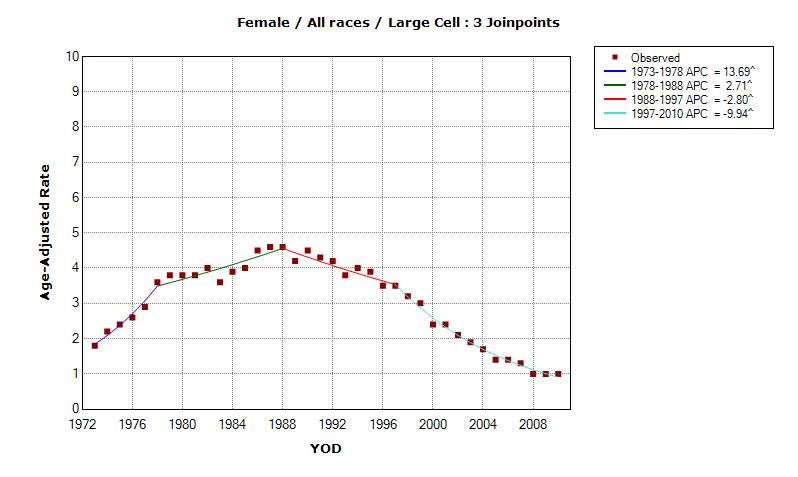

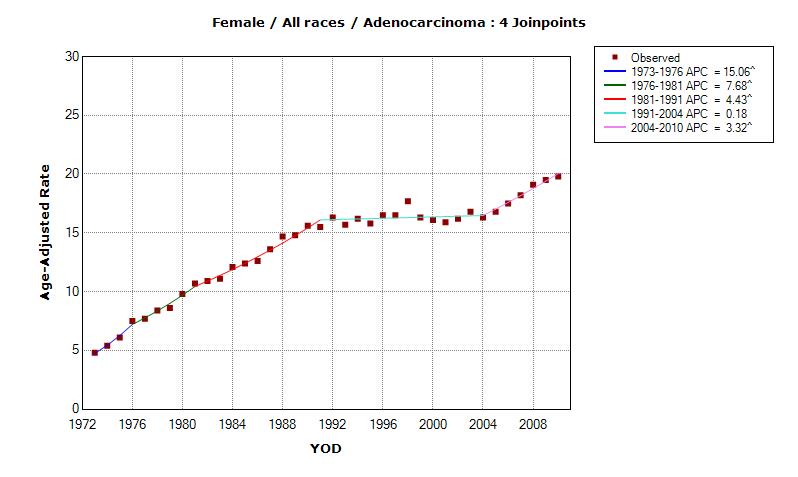

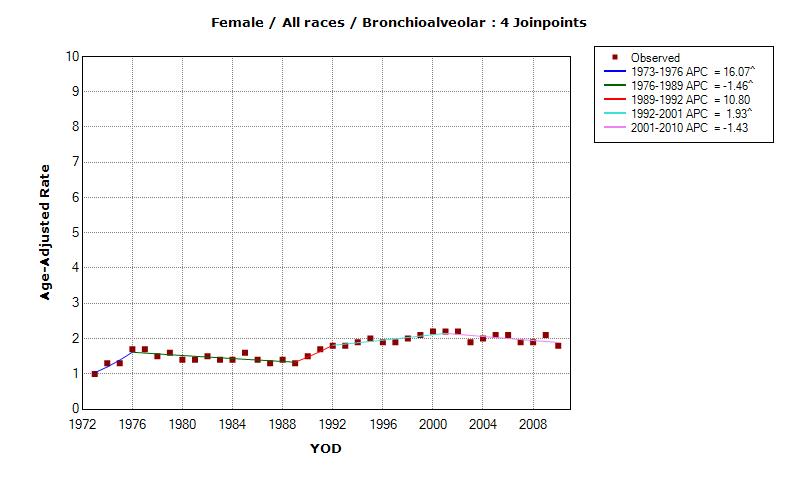


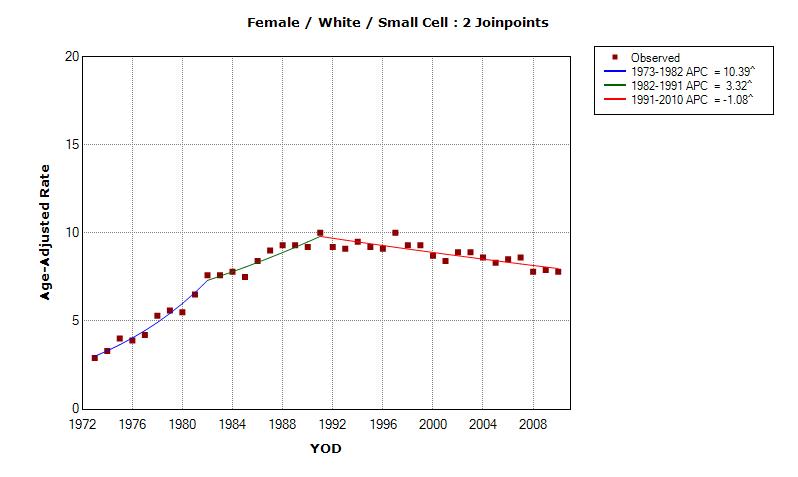

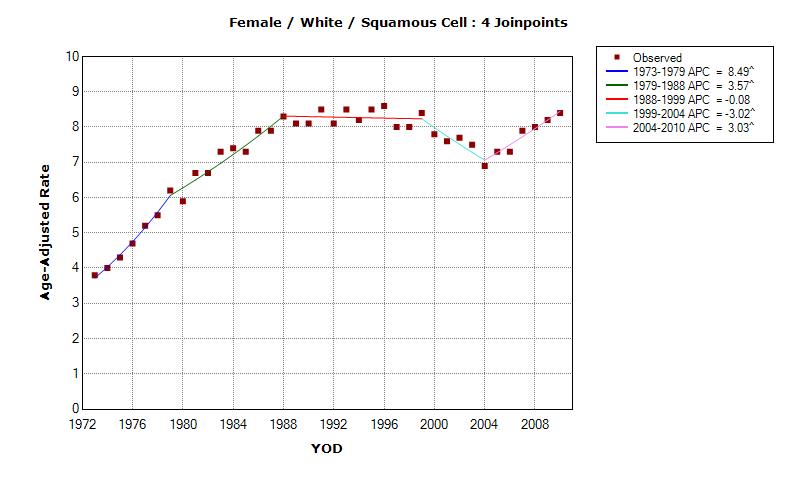

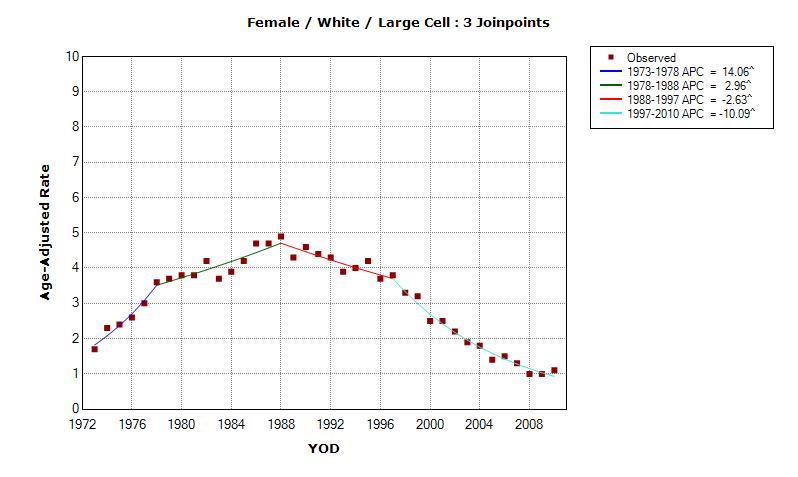

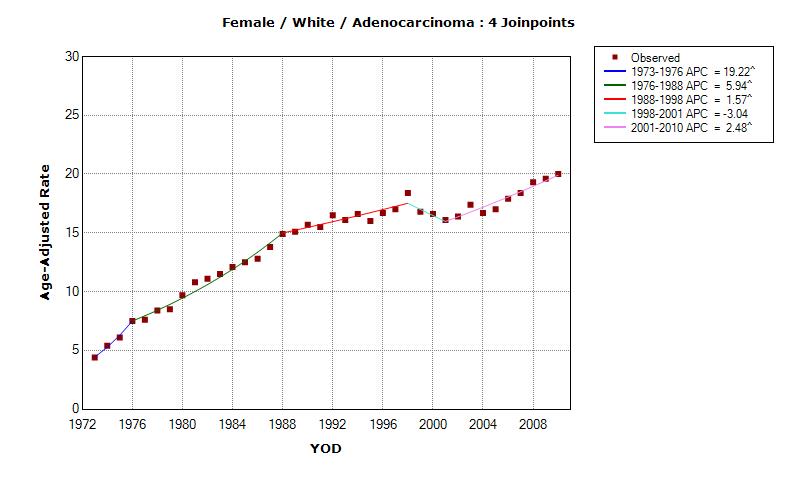

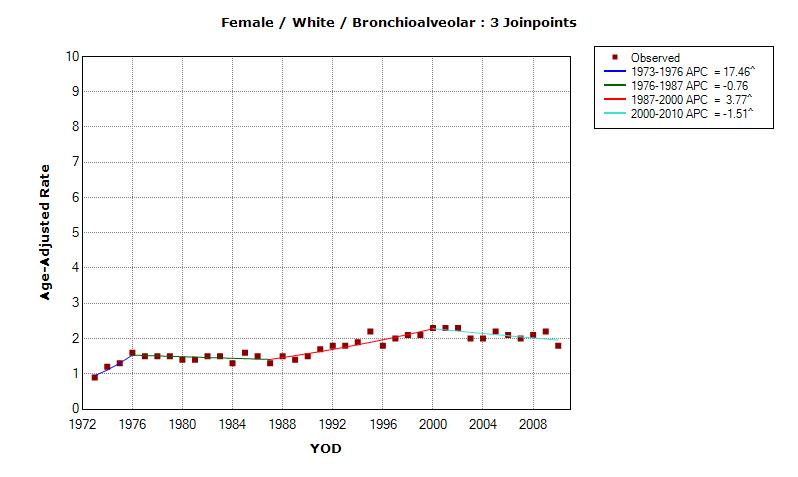

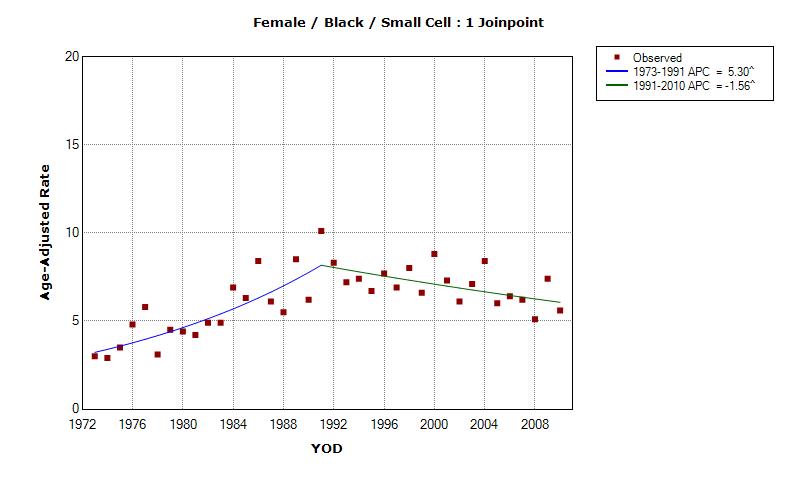

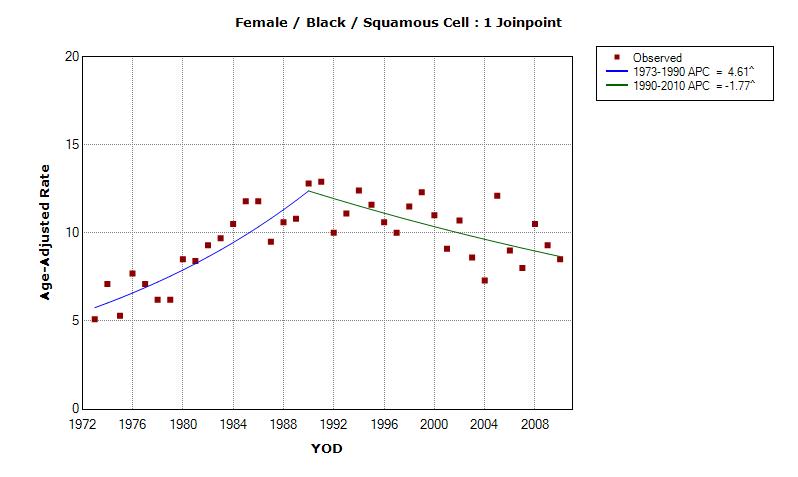

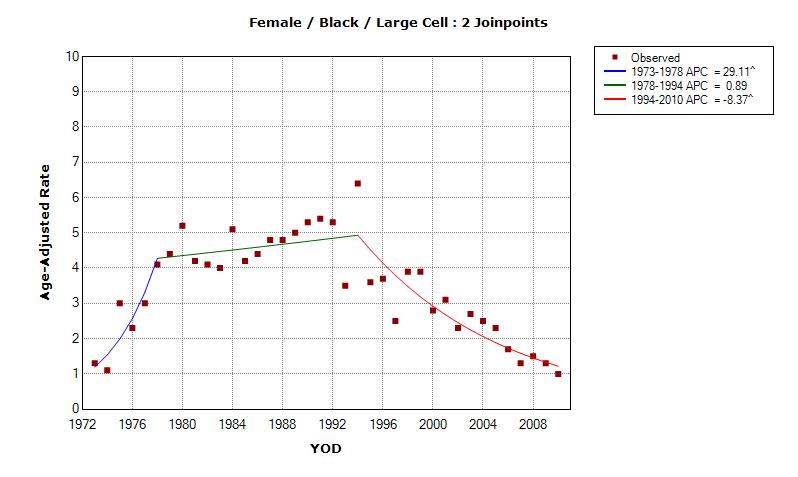

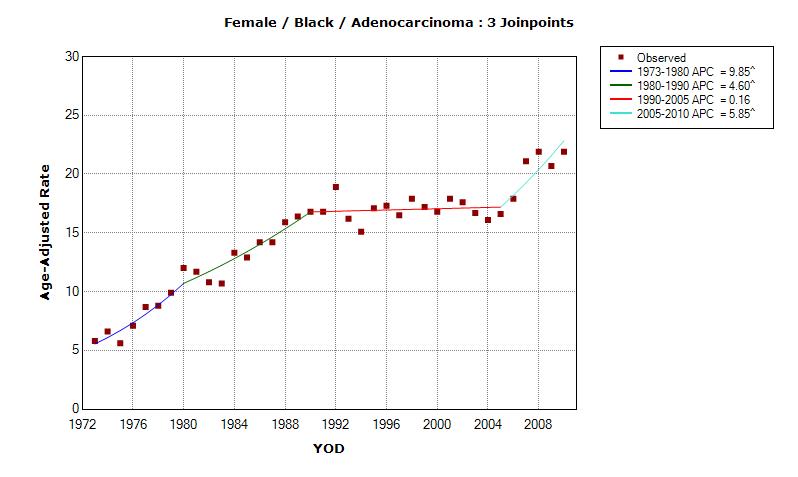

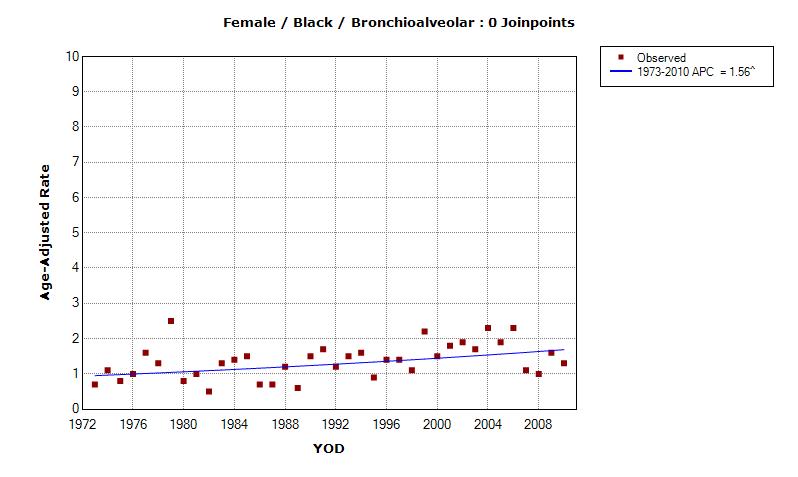


**ALL HISTOLOGIES**


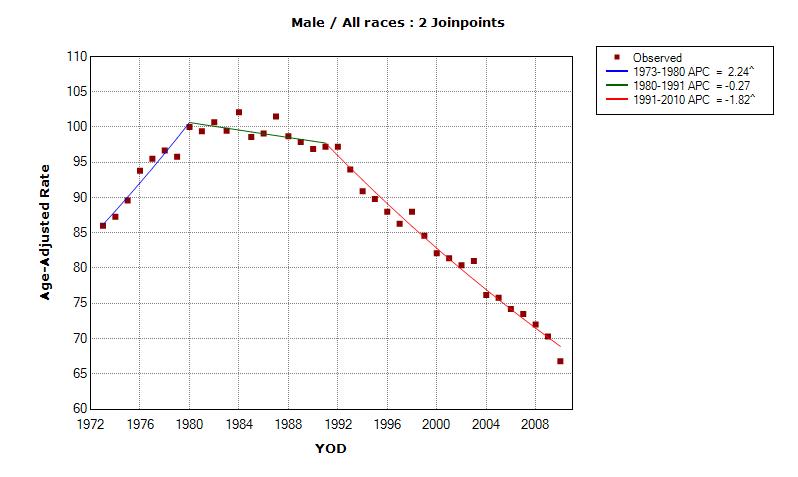

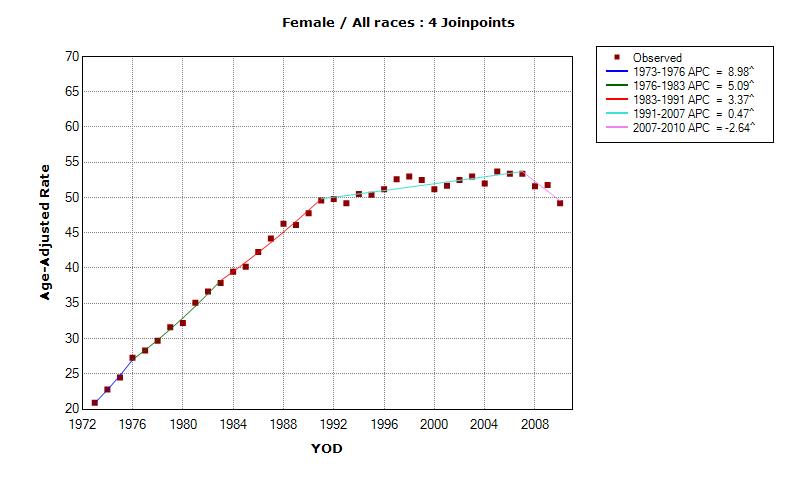


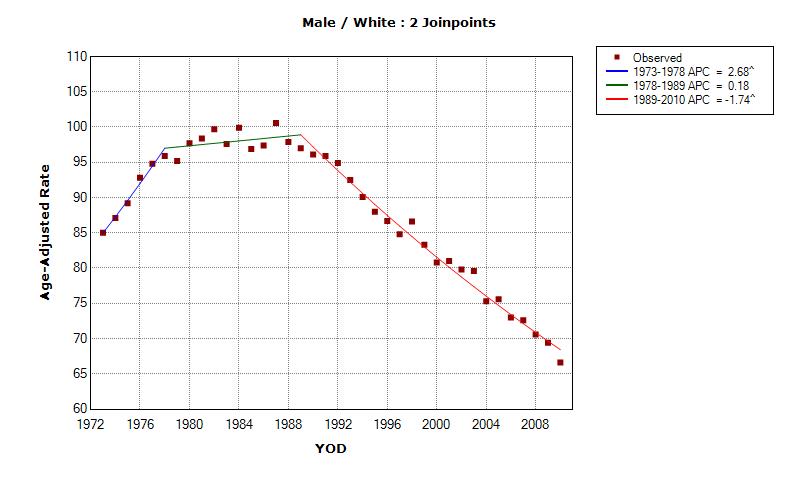

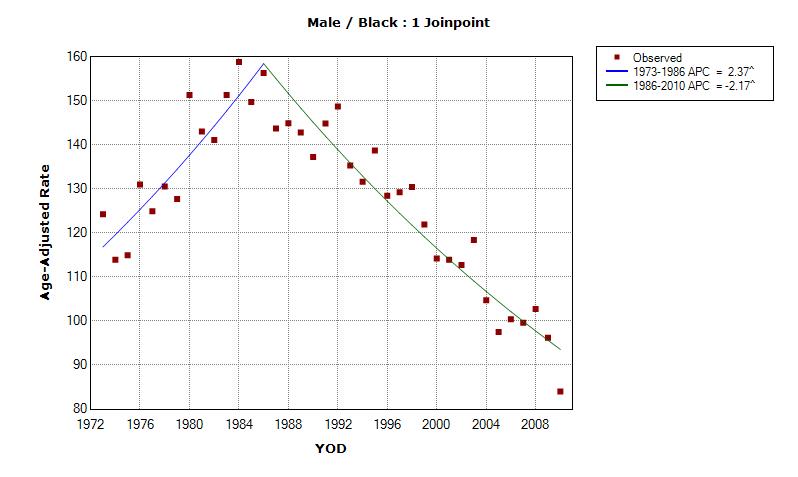


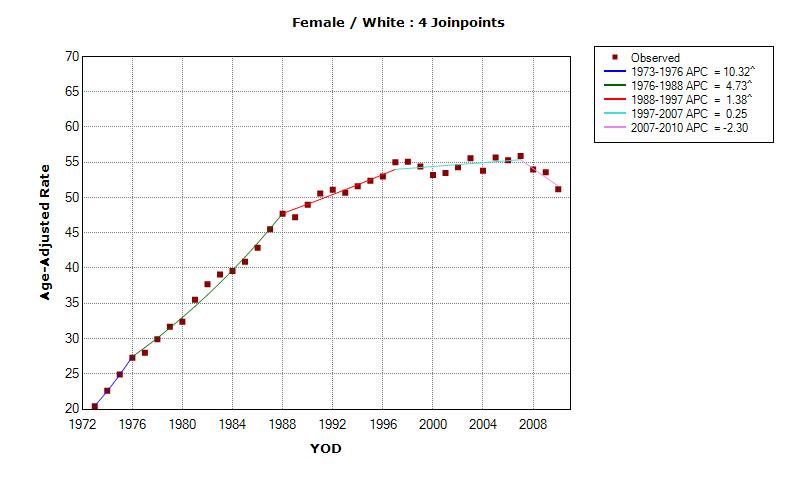

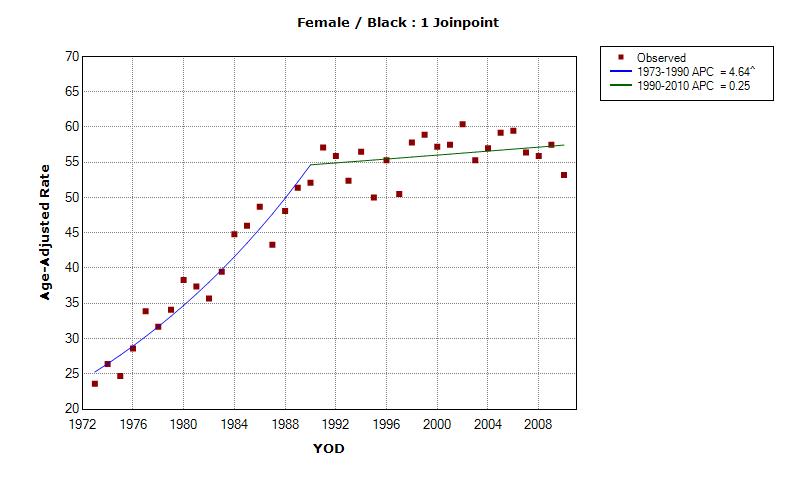

Supplement: S3 Fig — APC based on incidence rates per 100,000 person-years (^ indicates APC is significantly different from zero at α = 0.05). (DOC) [file pone.0121323.s003.doc]

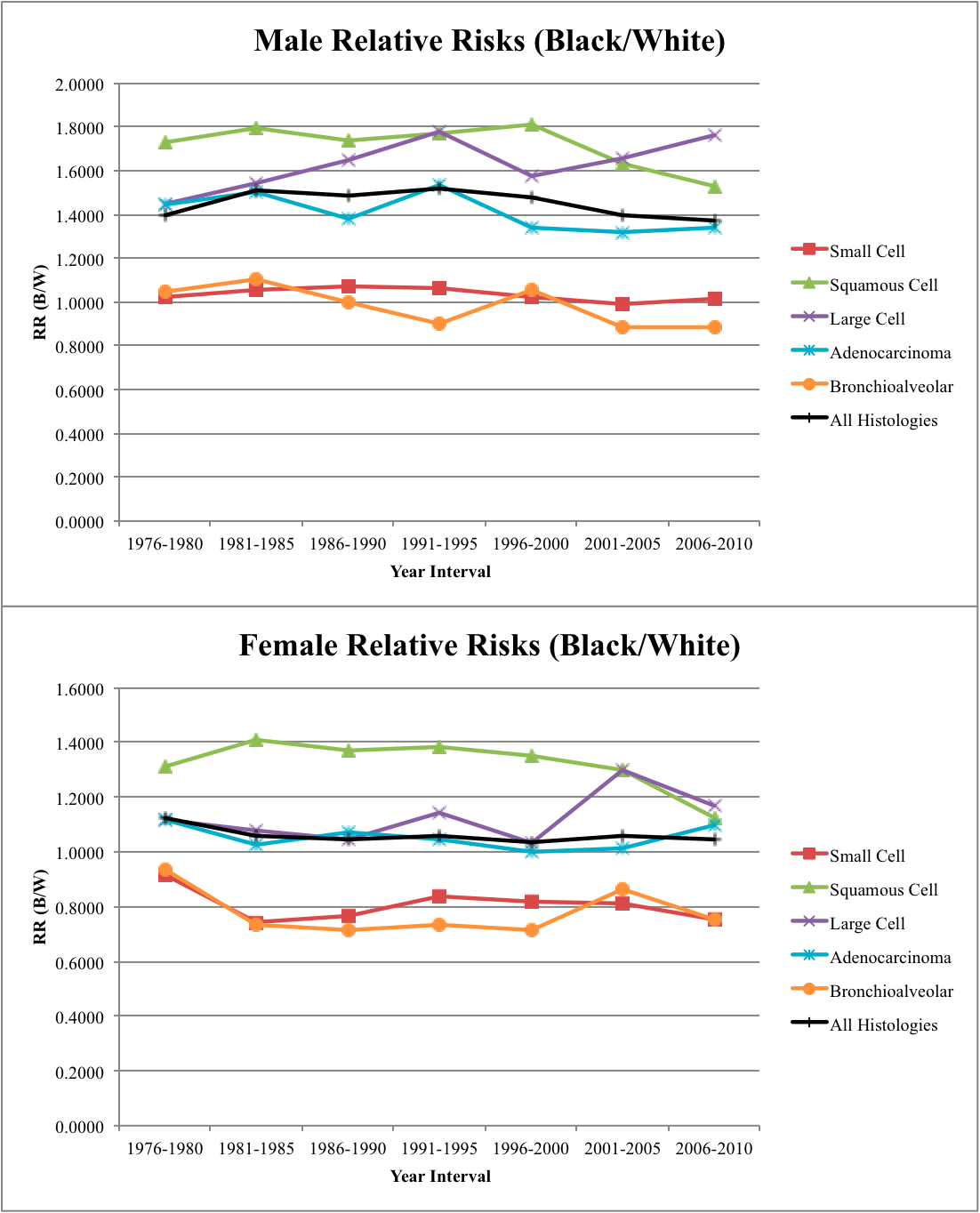

Supplement: S4 Fig — (TIFF) [file pone.0121323.s004.tiff]

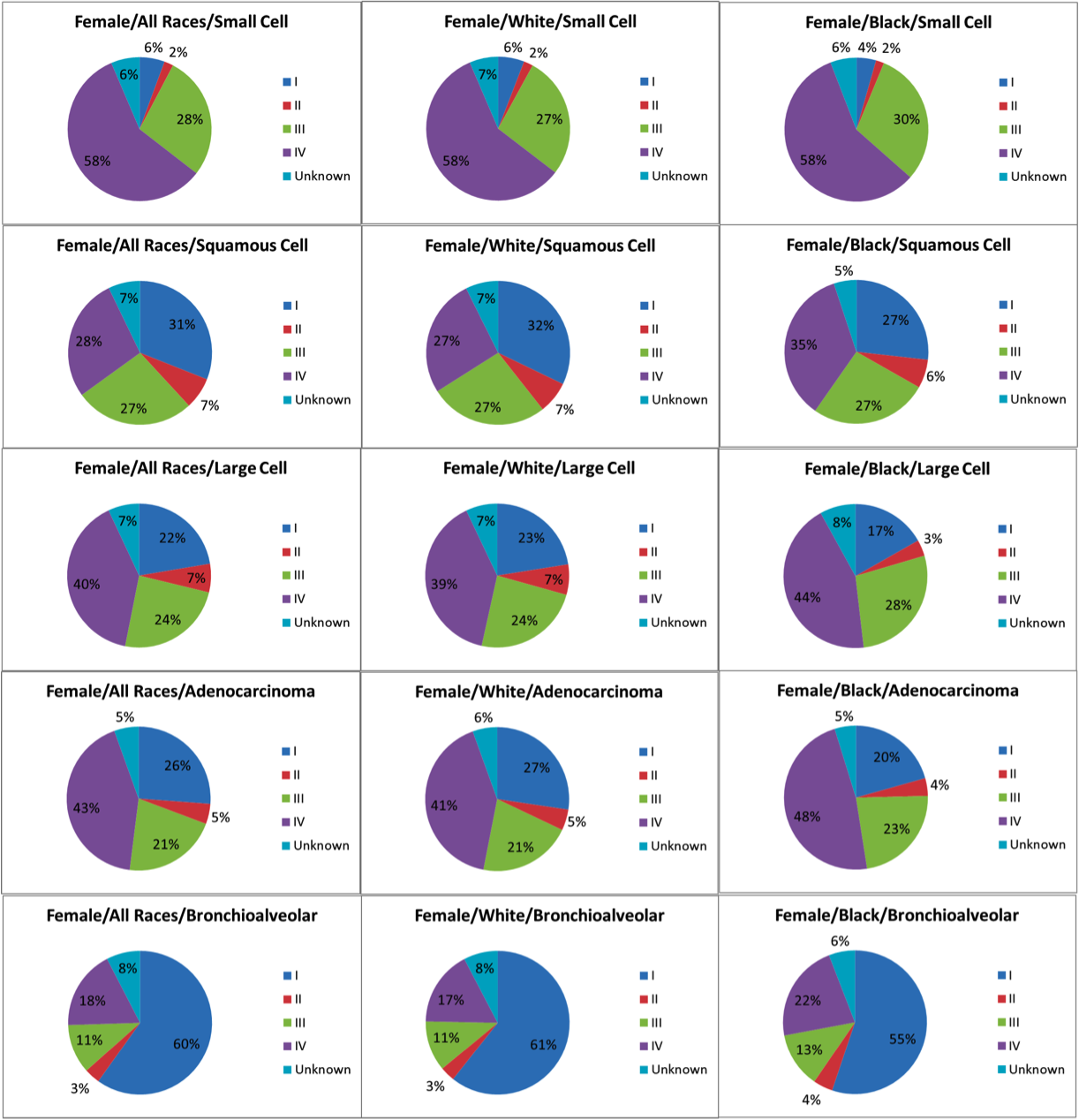

Supplement: S5 Fig — (TIFF) [file pone.0121323.s005.tiff]
