# Supplementary material for: Lung Cancer Incidence Trends by Gender, Race and Histology in the United States, 1973–2010
Source: PLoS One. 2015 Mar 30;10(3):e0121323. doi: 10.1371/journal.pone.0121323 (PMC4379166; doi:10.1371/journal.pone.0121323)
Supplement: S1 Table — aRate per 100,000 person-years (US Standard Population at year 2000) with 95% confidence intervals obtained using the Tiwari method. Rows are grouped by roughly 10-year time periods. (DOC) [file pone.0121323.s006.doc]

Table S1. Age-adjusted incidence rates^a^ (AAIR) of all lung and bronchus cancer cases reported to the United States SEER 9 registry by race and histology, 1973-2010

| **Male/All Races** |  | **Small Cell** | | **Squamous Cell** | | **Large Cell** | | **Adenocarcinoma** | | **Bronchioloalveolar** | | **All Histologies** | | |
| --- | --- | --- | --- | --- | --- | --- | --- | --- | --- | --- | --- | --- | --- | --- |
|  | **Person-Years** | **Cases** | **AAIR** | **Cases** | **AAIR** | **Cases** | **AAIR** | **Cases** | **AAIR** | **Cases** | **AAIR** | **Cases** | | **AAIR** |
| **1973-1985** | 132,631,315 | 13,986 | 13.1 (12.9, 13.4) | 32,452 | 31.4 (31, 31.7) | 10,262 | 9.7 (9.5, 9.9) | 19,233 | 18.1 (17.9, 18.4) | 2,154 | 2 (2, 2.1) | 97,926 | | 96.3 (95.6, 96.9) |
| **1986-1997** | 142,482,603 | 15,929 | 13.6 (13.4, 13.8) | 29,819 | 25.9 (25.6, 26.2) | 9,683 | 8.3 (8.1, 8.5) | 27,964 | 23.9 (23.6, 24.2) | 2,172 | 1.9 (1.8, 2) | 107,674 | | 94.5 (93.9, 95.1) |
| **1998-2010** | 176,566,728 | 15,004 | 9.6 (9.5, 9.8) | 25,580 | 17 (16.8, 17.2) | 4,685 | 3 (2.9, 3.1) | 33,432 | 21.7 (21.5, 21.9) | 2,564 | 1.7 (1.6, 1.8) | 116,482 | | 76.9 (76.4, 77.3) |
| **Male/White** |  | **Small Cell** | | **Squamous Cell** | | **Large Cell** | | **Adenocarcinoma** | | **Bronchioloalveolar** | | **All Histologies** | | |
|  | **Person-Years** | **Cases** | **AAIR** | **Cases** | **AAIR** | **Cases** | **AAIR** | **Cases** | **AAIR** | **Cases** | **AAIR** | **Cases** | **AAIR** | |
| **1973-1985** | 111,214,269 | 12,466 | 13.4 (13.2, 13.7) | 27,336 | 30.4 (30, 30.7) | 8,779 | 9.6 (9.3, 9.8) | 16,390 | 17.7 (17.5, 18) | 1,852 | 2 (1.9, 2.1) | 84,276 | 95.1 (94.4, 95.7) | |
| **1986-1997** | 113,930,554 | 13,904 | 14 (13.8, 14.2) | 24,610 | 25.2 (24.9, 25.6) | 7,958 | 8.1 (7.9, 8.3) | 23,177 | 23.4 (23.1, 23.7) | 1,829 | 1.9 (1.8, 2) | 89,988 | 93.3 (92.6, 93.9) | |
| **1998-2010** | 134,450,043 | 12,795 | 10 (9.9, 10.2) | 20,683 | 16.7 (16.5, 16.9) | 3,761 | 3 (2.9, 3.1) | 26,701 | 21.2 (21, 21.5) | 2,109 | 1.7 (1.6, 1.8) | 94,338 | 76 (75.5, 76.5) | |
| **Male/Black** |  | **Small Cell** | | **Squamous Cell** | | **Large Cell** | | **Adenocarcinoma** | | **Bronchioloalveolar** | | **All Histologies** | | |
|  | **Person-Years** | **Cases** | **AAIR** | **Cases** | **AAIR** | **Cases** | **AAIR** | **Cases** | **AAIR** | **Cases** | **AAIR** | **Cases** | **AAIR** | |
| **1973-1985** | 12,610,039 | 1,072 | 14 (13.1, 14.9) | 4,028 | 54 (52.2, 55.8) | 1,051 | 13.6 (12.7, 14.5) | 2,027 | 25.7 (24.5, 26.9) | 161 | 2.1 (1.8, 2.5) | 10,167 | 137.2 (134.3, 140.1) | |
| **1986-1997** | 15,436,330 | 1,286 | 14.7 (13.9, 15.6) | 3,778 | 44.7 (43.3, 46.2) | 1,240 | 13.5 (12.7, 14.3) | 3,052 | 33.6 (32.4, 34.9) | 167 | 1.9 (1.6, 2.2) | 11,998 | 139.5 (136.9, 142.2) | |
| **1998-2010** | 21,220,825 | 1,269 | 10.1 (9.5, 10.7) | 3,135 | 26.9 (25.9, 27.9) | 658 | 4.9 (4.5, 5.3) | 3,694 | 28.2 (27.2, 29.2) | 189 | 1.5 (1.3, 1.7) | 13,033 | 106 (104.1, 108) | |

^a^Rate per 100,000 person-years (US Standard Population at year 2000) with 95% confidence intervals obtained using the Tiwari method. Rows are grouped by roughly 10-year time periods.
