# Supplementary material for: Lung Cancer Incidence Trends by Gender, Race and Histology in the United States, 1973–2010
Source: PLoS One. 2015 Mar 30;10(3):e0121323. doi: 10.1371/journal.pone.0121323 (PMC4379166; doi:10.1371/journal.pone.0121323)
Supplement: S2 Table — aRate per 100,000 person-years (US Standard Population at year 2000) with 95% confidence intervals obtained using the Tiwari method. Rows are grouped by roughly 10-year time periods. (DOC) [file pone.0121323.s007.doc]

Table S2. Age-adjusted incidence rates^a^ (AAIR) of all lung and bronchus cancer cases reported to the United States SEER 9 registry by race and histology, 1973-2010

| **Female/All Races** |  | **Small Cell** | | **Squamous Cell** | | **Large Cell** | | **Adenocarcinoma** | | **Bronchioloalveolar** | | **All Histologies** | |
| --- | --- | --- | --- | --- | --- | --- | --- | --- | --- | --- | --- | --- | --- |
|  | **Person-Years** | **Cases** | **AAIR** | **Cases** | **AAIR** | **Cases** | **AAIR** | **Cases** | **AAIR** | **Cases** | **AAIR** | **Cases** | **AAIR** |
| **1973-1985** | 138,485,093 | 7,218 | 5.4 (5.3, 5.5) | 7,887 | 5.9 (5.8, 6.1) | 4,351 | 3.3 (3.2, 3.4) | 11,752 | 9.1 (8.9, 9.3) | 1,915 | 1.4 (1.4, 1.5) | 41,719 | 31.8 (31.5, 32.2) |
| **1986-1997** | 148,216,644 | 12,885 | 8.7 (8.5, 8.8) | 12,295 | 8.2 (8, 8.3) | 6,046 | 4.1 (4, 4.2) | 22,572 | 15.3 (15.1, 15.6) | 2,509 | 1.7 (1.6, 1.8) | 72,243 | 48.5 (48.1, 48.8) |
| **1998-2010** | 182,060,197 | 14,830 | 7.7 (7.6, 7.9) | 14,296 | 7.4 (7.3, 7.6) | 3,434 | 1.8 (1.7, 1.8) | 33,679 | 17.4 (17.2, 17.6) | 3,898 | 2 (2, 2.1) | 101,317 | 52 (51.7, 52.3) |
| **Female/White** |  | **Small Cell** | | **Squamous Cell** | | **Large Cell** | | **Adenocarcinoma** | | **Bronchioloalveolar** | | **All Histologies** | |
|  | **Person-Years** | **Cases** | **AAIR** | **Cases** | **AAIR** | **Cases** | **AAIR** | **Cases** | **AAIR** | **Cases** | **AAIR** | **Cases** | **AAIR** |
| **1973-1985** | 115,603,439 | 6,611 | 5.6 (5.5, 5.8) | 6,880 | 5.9 (5.7, 6) | 3,836 | 3.4 (3.3, 3.5) | 10,274 | 9.1 (8.9, 9.2) | 1,658 | 1.4 (1.4, 1.5) | 36,926 | 32 (31.7, 32.4) |
| **1986-1997** | 117,415,968 | 11,606 | 9.3 (9.1, 9.4) | 10,539 | 8.2 (8.1, 8.4) | 5,278 | 4.3 (4.1, 4.4) | 19,294 | 15.6 (15.4, 15.8) | 2,145 | 1.7 (1.6, 1.8) | 62,839 | 49.8 (49.4, 50.2) |
| **1998-2010** | 136,358,240 | 13,061 | 8.5 (8.3, 8.6) | 12,014 | 7.7 (7.6, 7.9) | 2,895 | 1.9 (1.8, 1.9) | 27,553 | 17.7 (17.5, 17.9) | 3,249 | 2.1 (2, 2.2) | 84,978 | 54.1 (53.7, 54.4) |
| **Female/Black** |  | **Small Cell** | | **Squamous Cell** | | **Large Cell** | | **Adenocarcinoma** | | **Bronchioloalveolar** | | **All Histologies** | |
|  | **Person-Years** | **Cases** | **AAIR** | **Cases** | **AAIR** | **Cases** | **AAIR** | **Cases** | **AAIR** | **Cases** | **AAIR** | **Cases** | **AAIR** |
| **1973-1985** | 13,805,817 | 468 | 4.7 (4.3, 5.1) | 791 | 8.2 (7.6, 8.8) | 361 | 3.7 (3.3, 4.1) | 948 | 9.8 (9.2, 10.5) | 112 | 1.2 (1, 1.5) | 3,336 | 35.2 (34, 36.5) |
| **1986-1997** | 17,046,680 | 910 | 7.4 (7, 7.9) | 1,354 | 11.2 (10.6, 11.8) | 552 | 4.5 (4.1, 4.9) | 1,978 | 16.3 (15.6, 17) | 147 | 1.2 (1, 1.4) | 6,271 | 51.9 (50.6, 53.2) |
| **1998-2010** | 23,387,602 | 1,187 | 6.8 (6.4, 7.2) | 1,644 | 9.7 (9.3, 10.2) | 407 | 2.2 (2, 2.4) | 3,333 | 18.6 (18, 19.3) | 292 | 1.7 (1.5, 1.9) | 10,005 | 57.2 (56, 58.3) |

^a^Rate per 100,000 person-years (US Standard Population at year 2000) with 95% confidence intervals obtained using the Tiwari method. Rows are grouped by roughly 10-year time periods.
